# IRF1 as a potential biomarker in *Mycobacterium tuberculosis* infection

**DOI:** 10.1111/jcmm.16756

**Published:** 2021-07-02

**Authors:** Liwei Wu, Qiliang Cheng, Zilu Wen, Yanzheng Song, Yijun Zhu, Lin Wang

**Affiliations:** ^1^ Department of Thoracic Surgery Shanghai Public Health Clinical Center Fudan University Shanghai China; ^2^ Department of Thoracic Surgery Tuberculosis Hospital of Shaanxi Province Xi’an China; ^3^ Department of Scientific Research Shanghai Public Health Clinical Center Fudan University Shanghai China; ^4^ TB Center Shanghai Emerging & Re‐emerging Infectious Diseases Institute Shanghai China

**Keywords:** biomarker, differentially expressed gene (DEG), network analysis, protein, protein interaction, tuberculosis (TB)

## Abstract

Pulmonary tuberculosis (PTB) is a major global public health problem. The purpose of this study was to find biomarkers that can be used to diagnose tuberculosis. We used four NCBI GEO data sets to conduct analysis. Among the four data sets, GSE139825 is lung tissue microarray, and GSE83456, GSE19491 and GSE50834 are blood microarray. The differential genes of GSE139825 and GSE83456 were 68 and 226, and intersection genes were 11. Gene ontology (GO) analyses of 11 intersection genes revealed that the changes were mostly enriched in regulation of leucocyte cell‐cell adhesion and regulation of T‐cell activation. Kyoto Encyclopedia of Genes and Genomes (KEGG) enrichment analysis of DEGs revealed that the host response in TB strongly involves cytokine‐cytokine receptor interactions and folate biosynthesis. In order to further narrow the range of biomarkers, we used protein‐protein interaction to establish a hub gene network of two data sets and a network of 11 candidate genes. Eventually, IRF1 was selected as a biomarker. As validation, IRF1 levels were shown to be up‐regulated in patients with TB relative to healthy controls in data sets GSE19491 and GSE50834. Additionally, IRF1 levels were measured in the new patient samples using ELISA. IRF1 was seen to be significantly up‐regulated in patients with TB compared with healthy controls with an AUC of 0.801. These results collectively indicate that IRF1 could serve as a new biomarker for the diagnosis of pulmonary tuberculosis.

## 1 INTRODUCTION

Tuberculosis is an infectious disease caused by *Mycobacterium tuberculosis*. Its main clinical features are prolonged low fever, expectoration and haemoptysis. *Mycobacterium tuberculosis* can infect tissues and organs all over the body, including lungs, intestines, lymph nodes, joints, spine and genitourinary system.^1^ Among them, pulmonary tuberculosis is the most common tuberculosis.

In recent years, tuberculosis has gradually become a public health problem that threatens human health. According to the tuberculosis report of the World Health Organization, about 10 million people in the world were infected with TB and 3 million died in 2018. It is also estimated that about 1/4 of the world's people are infected with *Mycobacterium tuberculosis*, which is at risk of developing tuberculosis.^2^ *Mycobacterium tuberculosis* is a kind of intracellular parasitic bacteria. Participation in immunity against *Mycobacterium tuberculosis* is mainly cellular immunity, including macrophages, T cells and NK cells.^3^

However, MTB can inhibit oxidative stress, apoptosis and autophagy, inhibit the synthesis of histocompatibility complex molecules and thus affect antigen presentation.^4^ It is precisely because of these mechanisms that inhibit the specific immunity and natural killing of macrophages, thus helping *Mycobacterium tuberculosis* to escape the immune killing of the body.^5^, ^6^ Therefore, a comprehensive view of the immune response mechanism of *Mycobacterium tuberculosis* infection has essential theoretical significance for clinical diagnosis and the study of novel tuberculosis vaccine and immunotherapy. The immune response in lung tissue can comprehensively reflect the lung immune response to *Mycobacterium tuberculosis*, so we can screen related molecular markers as sensitive indicators of tuberculosis infection. In this study, bioinformatics methods were used to compare and analyse the original genetic data of patients with pulmonary tuberculosis and healthy people, hoping to find the genes that may play an important role in the pathogenesis of tuberculosis, reveal the molecular immune mechanism of tuberculosis and discover the potential biomarkers of TB.

## 2 MATERIALS AND METHODS

### 2.1 Acquisition of RNA data

The data of human lung tissue samples and blood samples infected with TB were extracted from the GEO database.^7^ A total of 26 lung tissue samples were extracted from GSE139825,^8^ including 16 pulmonary tuberculosis (PTB) samples and 10 healthy control (HC) samples. A total of 70 blood samples were acquired from GSE83456, including 35 pulmonary tuberculosis (PTB) samples and 35 healthy control (HC) samples. The exclusion criteria were as follows: patients with immunodeficiency, such as HIV infection, congenital immunodeficiency; patients with hepatitis B; and patients with autoimmune diseases. All of the above conditions can affect pulmonary immunity, so they were excluded. The first verification data were extracted from GSE19491, including 21 PTB samples from London, 12 HC samples from London, 20 PTB samples from South Africa and 31 latent TB samples from South Africa. The second verification data were extracted from GSE50834, including 23 HIV‐infected samples and 21 HIV/TB co‐infected samples. All sample data were downloaded for further analysis. All sample data were from public databases, so informed consent and ethical approval were not required.

### 2.2 Identification of DEGs

The original expression matrix had been normalized by the uploader, so there was no need for normalized operation. The differentially expressed genes (DEG) were screened out by limma package.^9^ The *t* test method was used to calculate *P*‐value of genes, and the adjusted *P*‐value was calculated by Benjamini and Hochberg's method. The following selection criteria were used to screen out differentially expressed genes: ∣log FC∣> 1 between two sample groups; and adjusted *P*‐value <.05. Using Venn diagram, the DEGs of GSE139825 and GSE83456 were intersected.

### 2.3 Enrichment analysis

The Metascape (Metascape; https://metascape.org/gp/index.html/main#/step1) ^10^ is a database that provides a variety of online analysis tools. Its function is very powerful, can be used for enrichment analysis and can plot a relatively exquisite result map. Kyoto Encyclopedia of Genes and Genomes (KEGG) is a database that can analyse the uploaded gene list and find that the genes in the gene list are significantly enriched in certain pathways, so as to provide a reference for the function of genes.^11^ In addition, gene ontology (GO) analysis,^12^ including three annotations of biological processes (BPs), molecular functions (MFs) and cellular components (CCs), can be used to understand the function of genes in the uploaded gene list and to annotate genes. The Metascape database integrates the functions of KEGG and GO databases. The enrichment analyses were performed by the Metascape online database to analyse the function of DEGs. The false discovery rate (FDR) of results <0.05 was considered to be statistically significant.

### 2.4 Protein‐protein interaction network construction

We used the Search Tool for the Retrieval of Interacting Genes (STRING) online database (http://string‐db.org; version 11.0)^13^ to build the protein‐protein interaction (PPI) network. The mechanism of disease occurrence and development can be revealed by the functional interaction between proteins. In this study, we used the STRING database to build PPI networks of DEGs, and interactions with scores >0.4 were considered to be statistically significant. Cytoscape (version 3.8.0) is an open‐source software platform for visualizing complex networks and integrating these with any type of attribute data.^14^ CytoHubba^15^ is a Cytoscape plugin which was used to discover hub genes of PPI network. We used the maximum correlation criterion (MCC) in the CytoHubba plugin to screen 10 genes. The 10 genes were arranged according to the descending order of MCC scores, and the higher the order, the darker the redness of the genes.

### 2.5 Enzyme‐linked immunosorbent assay (ELISA)

The gene selected from the intersection between three gene lists was used as a biomarker for experimental verification. Therefore, we used 20 cases of pulmonary tuberculosis blood samples and 20 cases healthy control samples for verification. The patients were from the Shanghai Public Health Clinical Center. The candidate biomarkers were validated using the Human IRF1 kits (USCN Life Sciences; Wuhan, China). The experiment was carried out according to the manufacturer's instructions.

### 2.6 Verification of potential biomarker in verification data sets

Two data sets, GSE19491 and GSE50834, were used to evaluate the expression level of potential biomarker. We used idmap R package to find out potential biomarker expression level in two gene matrixes. The ggplot2 R package was used to draw box plot to show the different expression level in different groups. In GSE19491, there were three groups: HC groups, PTB groups and Latent TB groups. In GSE50834, there were two groups: HIV‐infected groups and HIV/TB co‐infected groups.

### 2.7 Gene Set Enrichment Analysis (GSEA) of two gene sets

Gene Set Enrichment Analysis (GSEA) is a computational method that determines whether an a priori defined set of genes shows statistically significant, concordant differences between two biological states (eg, phenotypes). We used the GSEA software to conduct the GSEA. P‐value <0.05 was statistically significant.

### 2.8 Statistical analysis

All statistical analysis was carried out by R (version: 3.6.2) software. Student t test was used to test the difference between the two groups. The receiver operating characteristic (ROC) curve was used to evaluate the reliability of candidate indicators as diagnostic biomarkers. If the area under curve (AUC) was greater than 0.8, it means that the reliability of the candidate biomarkers was higher. All P values were bilateral, and *P* < .05 was considered to be statistically significant.

## 3 RESULTS

### 3.1 Identification of DEGs in TB

**FIGURE 1:**
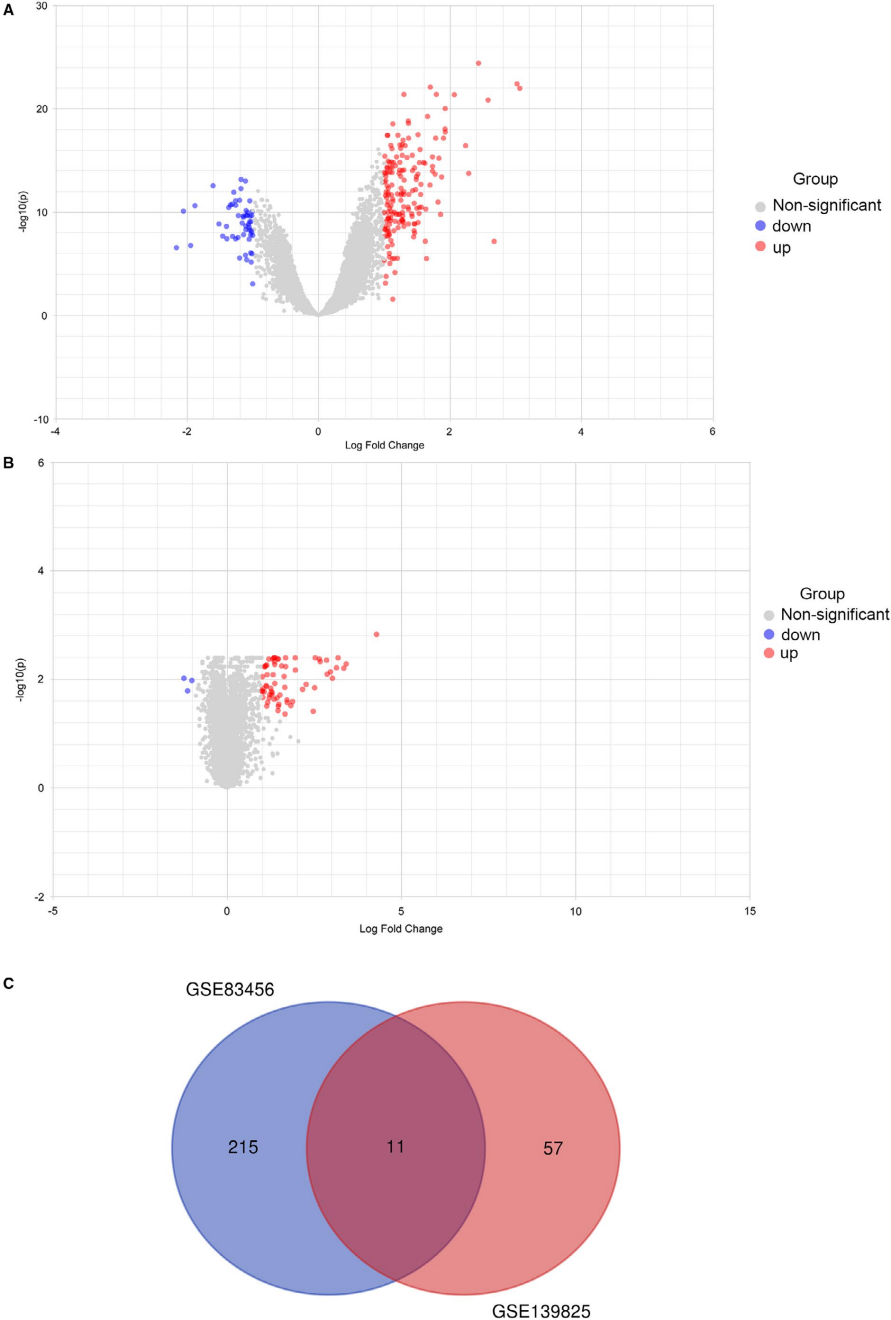
(A) Volcano plot demonstrated the distribution of GSE83456 differentially expressed genes, with a total of 225 genes, of which 173 genes are up‐regulated (red dots) and 52 genes are down‐regulated (blue dots). No significantly changed genes are marked as grey dots. B, Volcano plot demonstrated the distribution of GSE139825 differentially expressed genes, with a total of 68 genes, of which 65 genes are up‐regulated (red dots) and 3 genes are down‐regulated (blue dots). No significantly changed genes are marked as grey dots. C, Venn diagram of the intersection between DEGs of GSE139825 and GSE83456

Sixty eight DEGs were screened from the lung tissue gene expression matrix, among which 65 genes were up‐regulated and 3 genes were down‐regulated. In addition, 225 DEGs were screened from the blood gene expression matrix, among which 173 genes were up‐regulated and 52 genes were down‐regulated. Adjust *P* value <.05 and ∣logFC∣ >1 were defined as selection criteria. The intersection DEGs between GSE139825 and GSE83456 were as follows: IL7R, TNFAIP6, KLHDC8B, IRF1, HELZ2, ADM, CALHM6, GCH1, CD274, CCR7 and PSTPIP2. The volcano plot and Venn diagram were plotted based on the analysis results of the gene expression matrix (Figure 1).

### 3.2 Enrichment analyses of DEGs

**FIGURE 2:**
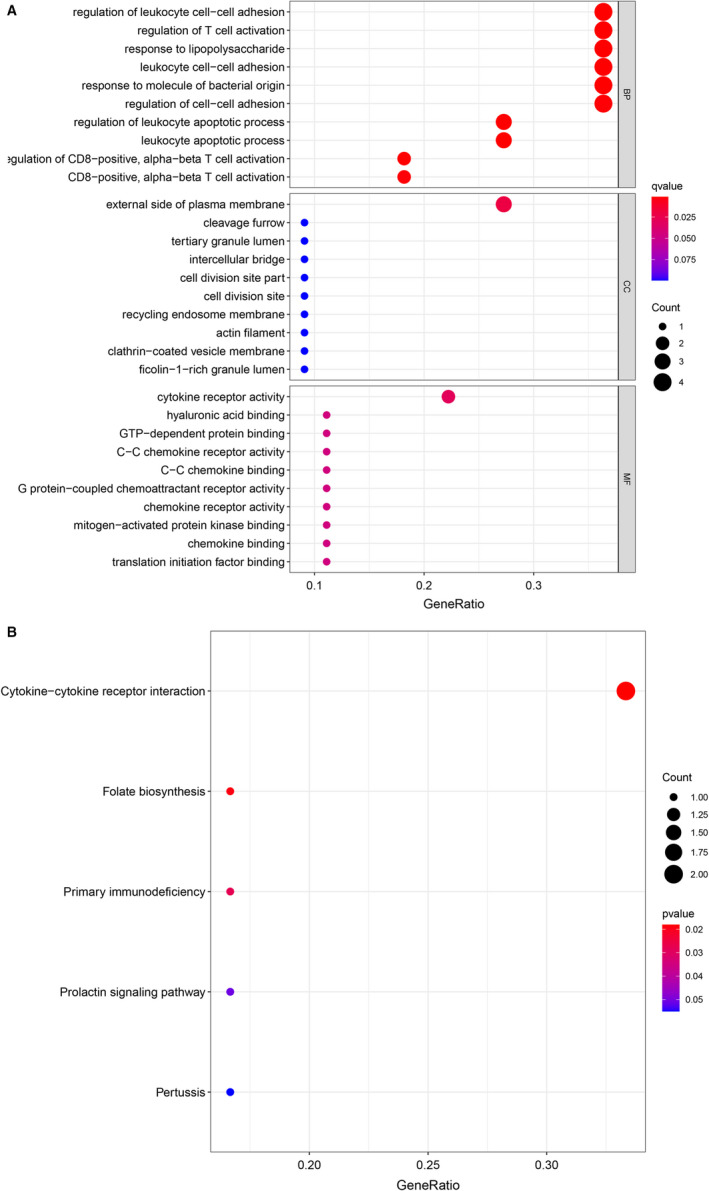
(A) GO and (B) KEGG pathway enrichment analysis of 11 intersection DEGs

Enrichment analysis was used to analyse the biological function of DEGs, and GO and KEGG pathway enrichment analyses of 11 intersection DEGs were performed (Figure 2). GO analysis results demonstrated that intersection DEGs were significantly enriched in regulation of leucocyte cell‐cell adhesion, regulation of T‐cell activation, response to lipopolysaccharide, leucocyte cell‐cell adhesion, response to molecule of bacterial origin, regulation of cell‐cell adhesion, external side of plasma membrane and cytokine receptor activity. KEGG pathway analysis demonstrated that the intersection DEGs were significantly enriched in cytokine‐cytokine receptor interaction, folate biosynthesis and primary immunodeficiency. The GO and KEGG enrichment analysis of DEGs in GSE139825 and GSE83456 were in Table 1 and Table 2.

**TABLE 1:** The GO and KEGG analysis of DEGs in GSE139825

Category	Description	Count	Log10(P)	Log10(q)
GO Biological Processes	response to lipopolysaccharide	26	−31.99	−27.62
GO Biological Processes	cellular response to interleukin‐1	18	−23.71	−20.13
GO Biological Processes	positive regulation of cytokine production	20	−19.21	−16.02
GO Biological Processes	defence response to other organism	21	−18.08	−15.04
GO Biological Processes	maintenance of location	14	−13.39	−10.72
GO Biological Processes	interleukin‐1 beta production	10	−12.98	−10.37
GO Biological Processes	positive regulation of MAPK cascade	16	−12.57	−9.99
GO Biological Processes	positive regulation of leucocyte migration	10	−11.73	−9.23
GO Biological Processes	regulation of multi‐organism process	13	−10.5	−8.1
GO Biological Processes	lipopolysaccharide‐mediated signalling pathway	7	−9.95	−7.61
GO Biological Processes	nitric oxide biosynthetic process	7	−9.04	−6.79
GO Biological Processes	response to mechanical stimulus	9	−8.69	−6.47
GO Biological Processes	regulation of interleukin‐2 production	6	−8.68	−6.46
GO Biological Processes	response to inorganic substance	12	−7.95	−5.83
GO Biological Processes	negative regulation of cytokine production	9	−6.56	−4.62
GO Cellular Components	external side of plasma membrane	10	−7.19	−5.15
GO Molecular Functions	CXCR chemokine receptor binding	4	−7.02	−5.01
KEGG Pathway	NOD‐like receptor signalling pathway	16	−20.73	−17.36
KEGG Pathway	Influenza A	10	−10.87	−8.44
KEGG Pathway	Transcriptional mis regulation in cancer	7	−6.55	−4.61

**TABLE 2:** The GO and KEGG analysis of DEGs in GSE83456

Category	Description	Count	Log10(P)	Log10(q)
GO Biological Processes	defence response to other organism	41	−25.22	−20.86
GO Biological Processes	regulation of innate immune response	35	−23.45	−19.39
GO Biological Processes	response to bacterium	34	−15.67	−12.31
GO Biological Processes	regulation of inflammatory response	27	−13.54	−10.22
GO Biological Processes	response to interferon‐gamma	18	−13.36	−10.07
GO Biological Processes	myeloid leucocyte activation	29	−13.03	−9.78
KEGG Pathway	Systemic lupus erythematosus	15	−12.72	−9.5
GO Biological Processes	regulation of immune effector process	23	−11.39	−8.26
GO Biological Processes	regulation of cell activation	25	−10.05	−7.23
KEGG Pathway	NOD‐like receptor signalling pathway	14	−10.04	−7.23
GO Biological Processes	regulation of response to biotic stimulus	13	−9.75	−7
GO Biological Processes	regulation of response to cytokine stimulus	14	−9.22	−6.54
GO Biological Processes	positive regulation of interferon‐beta production	7	−8.52	−5.9
GO Biological Processes	cytokine secretion	11	−8.06	−5.52
GO Biological Processes	response to lipopolysaccharide	16	−7.94	−5.41
GO Biological Processes	positive regulation of I‐kappa B kinase/NF‐kappa B signalling	12	−7.44	−5
KEGG Pathway	Pertussis	8	−6.8	−4.44
GO Biological Processes	response to interferon‐beta	6	−6.76	−4.4
GO Biological Processes	regulation of interleukin‐12 production	7	−6.59	−4.25
GO Biological Processes	response to tumour necrosis factor	14	−6.53	−4.21

### 3.3 PPI network construction and screening candidate biomarkers

**FIGURE 3:**
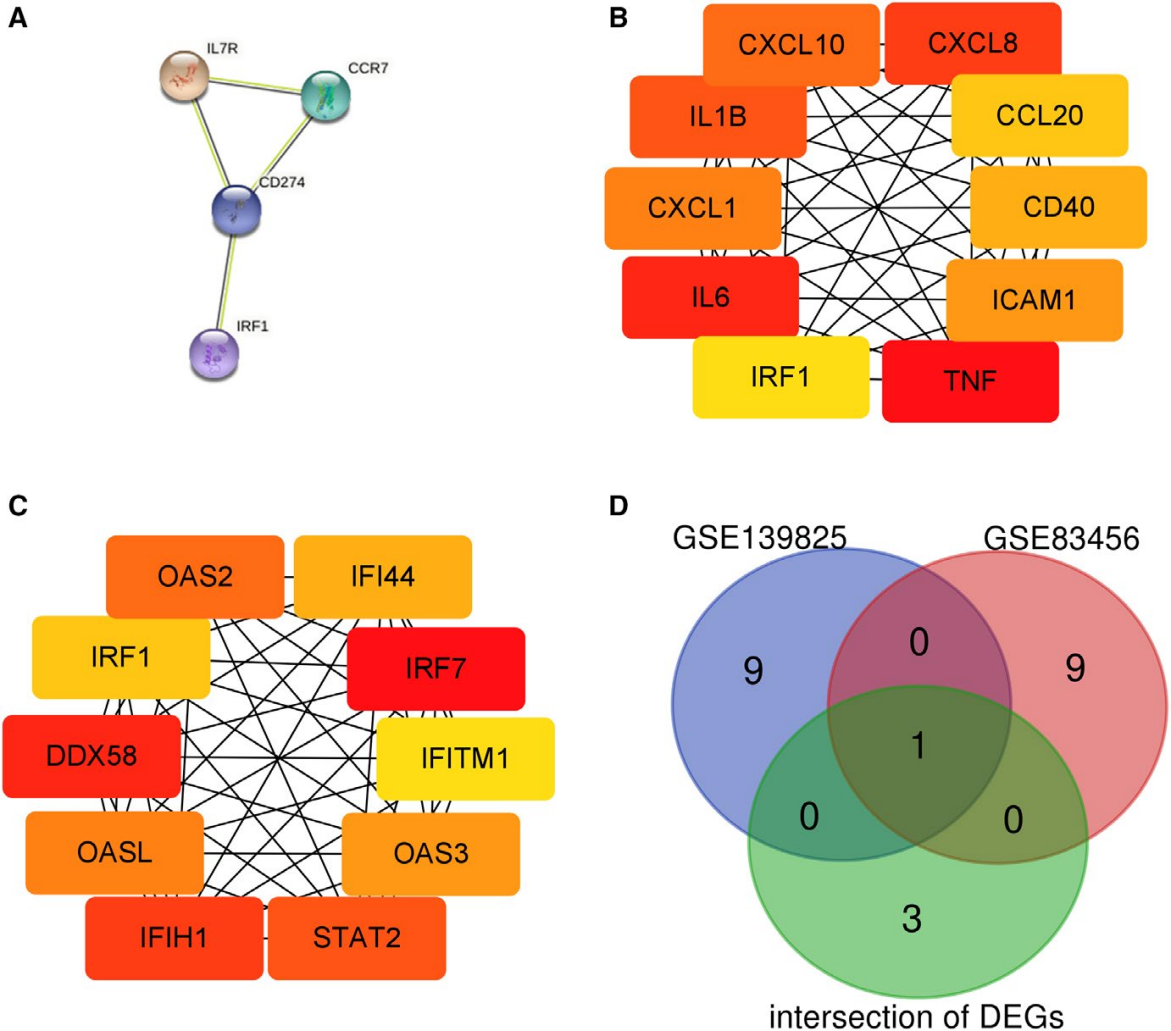
Tuberculosis (TB) PPI network. A, The interaction between 11 intersection DEGs; only four DEGs had the interaction. B, The interaction between hub genes of GSE139825. The darker the red, the higher the MCC ranking. C, The interaction between hub genes of GSE83456. The darker the red, the higher the MCC ranking. D, The Venn diagram of above three gene lists

We used STRING online database (version: 11.0) to analyse 11 intersection DEGs and obtained the interaction data of 11 intersection DEGs (Figure 3A). Then, 225 DEGs of GSE83456 and 68 DEGs of GSE139825 were also analysed. The data were exported as nodes for further analysis. Cytoscape software was used to visualize the obtained data. The cytoHubba plugin was used to analyse hub genes with MCC, and genes with the top 10 scores were identified as hub genes (Figure 3B, C). We took intersection between the hub genes of GSE83456 and GSE139825 and intersection DEGs. The IRF1 was the only one intersection gene (Figure 3D).

### 3.4 Verification of potential biomarker expression by ELISA

**FIGURE 4:**
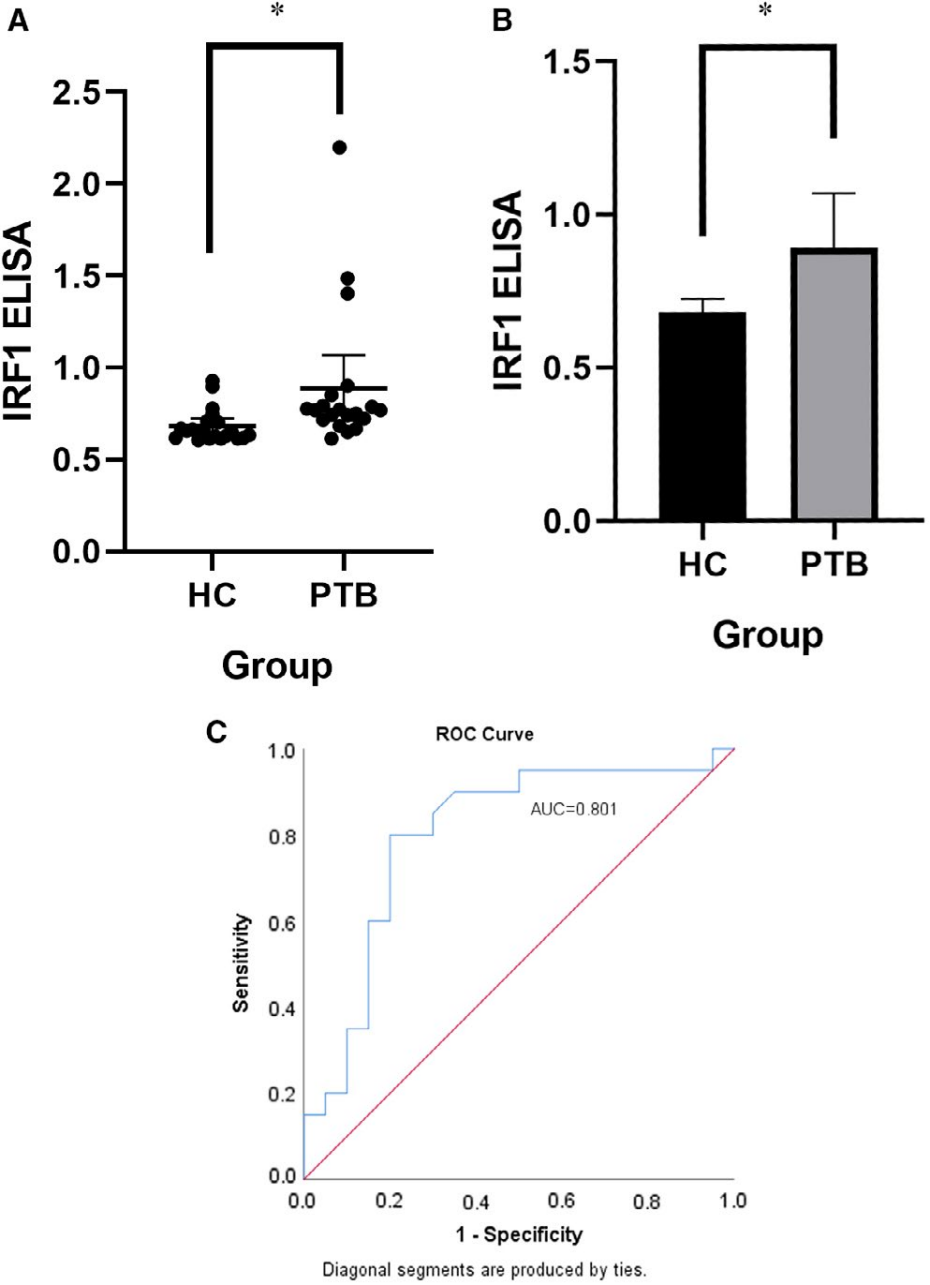
Enzyme‐linked immunosorbent assay (ELISA) results and ROC curve of IRF1. HC means healthy control. PTB means pulmonary tuberculosis

The IRF1 was selected as a candidate biomarker for experimental verification. The experimental results showed that IRF1 was up‐regulated in tuberculosis group, which was consistent with the results of our bioinformatics analysis (Figure 4A, B). The ROC curve showed an AUC of 0.801 (Figure 4C), demonstrating that IRF1 could be a diagnostic biomarker of PTB.

### 3.5 Verification of potential biomarker expression in verification data sets

**FIGURE 5:**
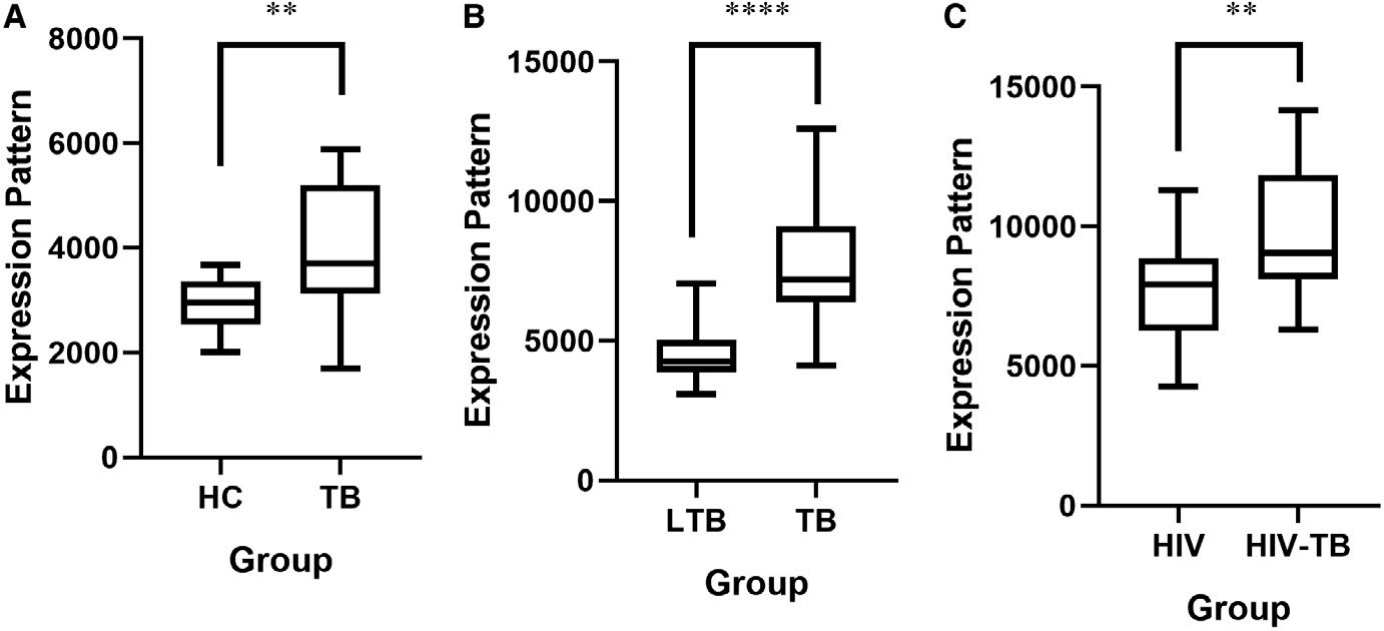
Verification of IRF1 in GSE19491 and GSE50834. A, The expression level of IRF1 was up‐regulated in TB group from HC group in GSE19491. B, The expression level of IRF1 was up‐regulated in TB group from LTB group in GSE19491. C, The expression level of IRF1 was up‐regulated in HIV/TB co‐infected group from HIV infected group in GSE50834

The IRF1 expression level in GSE19491 was as follows: IRF1 was up‐regulated in TB group from HC group (Figure 5A); and IRF1 was up‐regulated in TB group from Latent TB group (Figure 5B). The IRF1 expression level in GSE50834 was up‐regulated in HIV/TB‐co‐infected group from HIV‐infected group (Figure 5C). The above results were consistent with the results of our bioinformatics analysis.

### 3.6 Gene Set Enrichment Analysis (GSEA) of two gene sets

**FIGURE 6:**
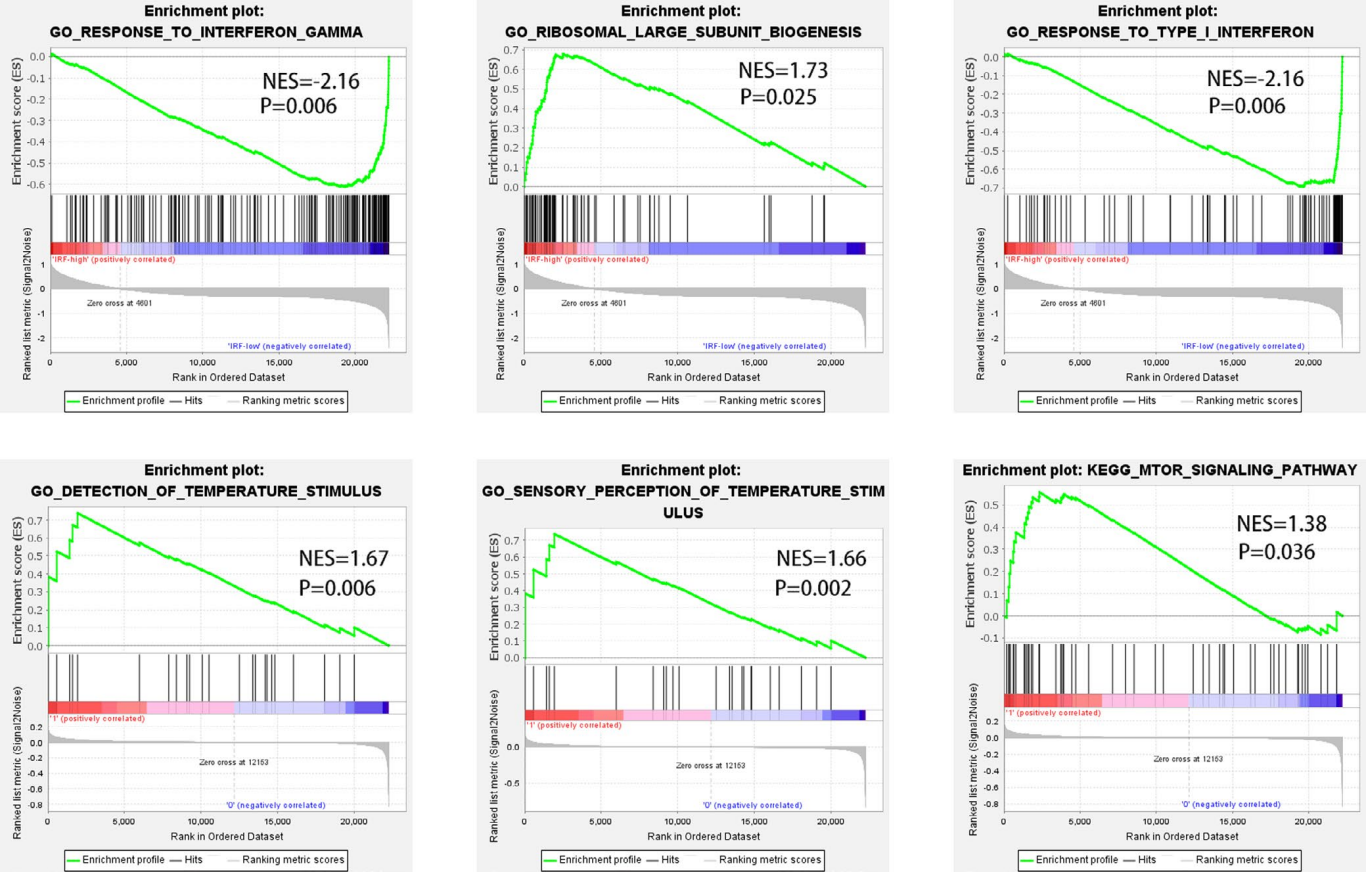
Gene Set Enrichment Analysis (GSEA) results of GSE83456 and GSE139825 IRF1 low expression group and IRF1 high expression group. The immunity of low group was different from that of high group

According to the amount of IRF1 expression, we divided the samples of the two data sets into two groups: IRF1 low expression group and IRF1 high expression group, and then analysed the two data sets by GSEA (Figure 6). According to the results of GSEA, most of the genes in the tissue samples of the high expression IRF1 group were enriched in the temperature regulation functional area, indicating that there was a higher inflammatory response in the focus of pulmonary tuberculosis. However, most of the genes in blood samples with high expression IRF1 are enriched in RNA translation‐related functions.

## 4 DISCUSSION

Tuberculosis has gradually become one of the diseases that threaten human health all over the world. It is caused by *Mycobacterium tuberculosis* parasite in macrophages. At present, a large number of data are based on the peripheral blood of patients with TB, but there are few data based on the lung tissue of patients with TB.^16^, ^17^, ^18^ Therefore, understanding the gene expression in lung tissue after tuberculosis infection can reveal the pathogenesis of tuberculosis and develop targeted treatment strategies. In this study, we used data form lung tissue microarray and blood microarray to find out the candidate biomarkers in PTB. After a series of analysis, the candidate biomarker was determined to be IRF1, and verified by ELISA and ROC curves. IRF1 may be used as a biomarker for the diagnosis of tuberculosis.

In order to find out the host response in the process of tuberculosis infection, we used Metascape online database to analyse biological process of 68 DEGs. Among these BP annotations, cellular response to lipopolysaccharide, positive regulation of leucocyte migration and defence response to other organism were considered to play a key role in tuberculosis immunity. Tuberculosis is characterized by the parasitism of *Mycobacterium tuberculosis* in macrophages and the use of macrophages for proliferation.^19^ At the same time, *Mycobacterium tuberculosis* will release endotoxin to induce macrophages to release cytokines and attract leucocyte, mainly neutrophils and T cells.^20^, ^21^ Leucocyte destroys macrophages and forms necrotic foci. Finally, coagulative necrotic areas were formed.^21^ This is consistent with the results of our data mining. However, in the blood data set, the functional enrichment of differential genes is different. GO enrichment analysis showed that the main function was enriched in innate immunity. Through the functional analysis of different genes in tissue and blood, it can be found that tissue and blood show different immune states.

Among the enriched signalling pathways in lung tissue and blood, three are considered to be significantly related to tuberculosis, including the NOD‐like receptor signalling pathway, Toll‐like receptor signalling pathway and TNF signalling pathway. At the same time, we found that these pathways are associated with idiopathic pulmonary fibrosis and lung cancer.^22^, ^23^ This may be the mechanism of advanced pulmonary fibrosis or lung cancer in some patients with TB. Meanwhile, these pathways are used to develop targeted drugs to block the proliferation of *Mycobacterium tuberculosis* in macrophages. This can shorten the time required for standardized TB treatment and improve the effectiveness of anti‐TB drugs.

Through GSEA analysis, we divided the samples into two groups: the increased expression of IRF1 and the decreased expression of IRF1. The results showed that the function of the gene was different between the high expression group of IRF1 and the low expression group of IRF1. This shows that IRF1 is a key gene in the occurrence and development of tuberculosis. It is expressed in blood and tissue and is suitable to be used as a biomarker for the diagnosis of tuberculosis. The specific function and mechanism of IRF1 need to be verified by further experiments.

In this study, we made a comprehensive bioinformatics analysis using the gene expression data of patients with TB. Although this study strongly predicted the potential genes and mechanisms involved in TB, it was not clear whether gene expression data based on public databases are reliable. Our approach improved our understanding of potential biomarkers for TB diagnose. However, our research also had some limitations. First of all, in order to fully clarify the molecular mechanism of the occurrence and development of TB, more gene chip samples of patients with TB were needed. Secondly, many biomarkers related to TB were still not characterized, and more experimental verification and bioinformatics analysis were needed to study the genes involved in tuberculosis. In the future, a prospective study may be needed to further study the biomarkers predicted in this study.

To conclude, our study finds out the IRF1 can be a potential biomarker for TB diagnose. Two gene sets GSE83456 and GSE139825 were analysed comprehensively. These results provided an updated perspective on the immune mechanism of tuberculosis and can be used for TB diagnosis.

## CONFLICT OF INTEREST

The authors declared that they have no conflict of interest.

## AUTHOR CONTRIBUTIONS

**Liwei Wu:** Formal analysis (lead); Writing‐original draft (lead); Writing‐review & editing (lead). **Yanzheng Song:** Data curation (equal); Funding acquisition (lead). **Lin Wang:** Methodology (equal); Software (equal). **Zilu Wen:** Data curation (equal); Formal analysis (equal). **Yijun Zhu:** Data curation (equal); Methodology (equal). **Qiliang Chen:** Data curation (equal); Funding acquisition (equal); Investigation (equal).

## Data Availability

The data that support the findings of this study are openly available in NCBI GEO database at https://www.ncbi.nlm.nih.gov/geo/
